# Jumping with control: the interplay between psychological constructs and run-up variability in elite jumpers

**DOI:** 10.3389/fpsyg.2024.1412910

**Published:** 2024-06-26

**Authors:** Larissa de Paula Moura, Nelio Alfano Moura, Tania Fernandes de Paula Moura, Túlio Bernardo Macedo Alfano Moura, Maria Regina Ferreira Brandão

**Affiliations:** ^1^Universidade São Judas Tadeu - Postgraduate Program in Physical Education, São Paulo, São Paulo, Brazil; ^2^Esporte Clube Pinheiros, São Paulo, São Paulo, Brazil; ^3^Centro de Excelência Esportiva – SESP, São Bernardo do Campo, São Paulo, Brazil; ^4^NAR – Nucleus of High Performance in Sport, São Paulo, Brazil

**Keywords:** athletics, long jump, triple jump, run-up variability, emotional regulation, selfcontrol

## Abstract

**Objectives:**

The purpose of this study was to examine the relationship between psychological aspects (emotional regulation, self-control, mood states, and perceived stress) and components of run-up variability in horizontal jumps and to conduct comparisons based on sex, events (long jump and triple jump), and contextual situations (training versus competition).

**Methods:**

A total of 10 elite-level athletes (five males and five females) with a mean age of 27.14 (±4.25) years were recruited for the study. All participants had competed nationally or internationally and had 13.10 (±3.48) years of athletic experience. Data were collected during competitions and training sessions for 5 weeks. The participants completed the Brunel Mood Scale, Emotional Regulation Questionnaire, Brief Self-Control Scale, and Visual Analogical Scale of Perceived Stress before each session. The components of run-up variability of successful and failed attempts were measured using video analysis. Data were analyzed using a t-test, Pearson’s correlation, and Cohen’s d.

**Results:**

Athletes specializing in long jump and triple jump displayed similar psychological and run-up variability characteristics. However, females showed higher values for tension and depression, whereas males had higher run-up speeds and vigor. In competitions, athletes tended to have higher vigor, lower fatigue and confusion, an earlier beginning of the adjustment phase, fewer failed attempts, and higher run-up speed than during training. Emotional regulation is inversely related to depression in women during competitions, whereas higher self-control is associated with fewer failed jumps.

**Conclusion:**

Athletes competing in the long jump and the triple jump do not differ in psychological traits and run-up characteristics, which suggests that similar training strategies can be used in both events. However, different solutions should be used considering the sex of athletes, with a particular focus on utilizing emotion regulation tools to modulate depression in female jumpers. It is recommended to include training sessions that simulate competition demands, primarily to ensure the early onset of the run-up adjustment phase.

## 1 Introduction

Long and triple jumps are two of the most demanding events in track and field, requiring speed, power, and technique. Success in these events heavily depends on the ability to execute a consistent and effective run-up, which is crucial for athletes to hit the board accurately and achieve the optimal speed and proper position to jump (Hay, 1998). However, run-up, which comprises programmed acceleration and adjustment phases, is also one of the most challenging aspects of these events, and even slight variations in speed or step length can significantly impact performance. Inconsistencies in step lengths during the run-up accumulate due to factors such as wind, surface type, and the athlete’s physical condition. Although athletes train to adjust the length of the last steps considering these factors, many failed attempts continue to occur (Wu et al., 2013).

Failed jumps can affect performance because they can alter the running pattern of subsequent attempts (McCosker et al., 2019), increase anxiety levels, and decrease confidence, impairing the athlete’s ability to execute the jump properly. The variability in run-up has been extensively studied owing to its biomechanical aspects and visual regulation (Lee et al., 1982; Hay, 1993; Scott et al., 1997; Panteli et al., 2011; Theodorou et al., 2011; Panteli et al., 2014; Makaruk et al., 2015; Panteli et al., 2015; Starzak et al., 2016). It has been shown that run-up variability follows a standard pattern, with inconsistencies accumulating during the acceleration phase and corrections beginning approximately five steps before the take-off board (Hay, 1993). Makaruk et al. (2015) found that the earlier adjustments to step length are made, the greater the speed conserved at take-off. However, these factors alone do not explain the individual strategies employed by athletes during competition (McCosker et al., 2021).

The visual system significantly influences a jumper’s performance as it is linked to the perception-action system (Miller and Clapp, 2011). However, athletes with low vision (category F13) regulated their step length in the same manner as sighted athletes, albeit with limited visual information (Theodorou et al., 2011). An experiment with F11 category athletes yielded equivalent results (Theodorou and Skordilis, 2012), suggesting that the ability to adjust the final steps to hit the board can also be influenced by kinesthetic or auditory feedback.

More experienced athletes have superior perceptual abilities and information processing capacity (Panteli et al., 2014), which may explain their better performance than less experienced athletes. Visual regulation influences run-up variability (Hay, 1998; Bradshaw and Aisbett, 2006; Panteli et al., 2011, 2014), although visual control has not been measured objectively in many studies. In Hildebrandt and Cañal-Bruland (2020) compared measures of visual regulation based on locomotion, as in the traditional method, with measures based on visualization among athletes wearing special glasses. They found that the beginning of visual regulation, determined by the locomotion method, coincided with the step where the longest gaze toward the take-off board was observed, but not with the step of the first visualization. Despite this pioneering study, the scarcity of objective measures calls attention to other aspects that may influence run-ups in horizontal jumps.

Despite the number of studies investigating the biomechanical aspects and visual regulation of run-ups, few have examined the psychological factors that may affect performance, highlighting an essential gap in our understanding of run-up variability. Scott et al. (1997), for example, found that the need to make valid attempts is one of the factors that affects run-up, suggesting that anxiety in training and competition contexts can influence its variability. Lee et al. (1982) also reported that run-up variability could be affected by factors such as confidence, fatigue, and wind. McCosker et al. asserted that the environment of horizontal jumping events is a complex system with different intervening variables, including psychological (McCosker et al., 2019) variables, that must be studied.

A recent study proposed that elite jumpers must adapt their actions to the competition’s physical and emotional demands (McCosker et al., 2021). Horizontal jumps are perceived as a series of connected events, and athletes must employ self-regulation strategies to achieve their performance goals. When considering the manifestations of psychological constructs during sports practice, it has been suggested that individuals seek to experience emotions that bring them closer to their goals, regardless of the pleasure or displeasure that these emotions may arouse (Tamir, 2009). Nonetheless, positive emotions such as happiness and excitement benefit concentration and performance (Vast et al., 2010), and emotional regulation strategies can be used to induce these states.

Emotional regulation plays a crucial role in an athlete’s performance by affecting their movement, reaction time, range of motion, and force production (Beatty et al., 2014). To optimize performance, athletes use emotional regulation strategies such as relaxation, attention redirection, self-talk, and imagery, which help create the best possible emotional climate (Lane et al., 2011, 2012). Among these strategies, self-talk is effective in improving concentration and performance, and has been used in preparation by international and Olympic athletes (Balague, 2000; Gould et al., 2001, 2005; Blumenstein and Lidor, 2007; Scala et al., 2018). Through emotional regulation, athletes can modify the intensity of their emotions to direct them toward desired levels.

Another aspect that has received significant attention from researchers is mood states. Mood and emotion are part of the same conceptual model, and a clear distinction between them is not always possible (Lane and Terry, 2000). In addition, mood and emotion are evaluated in the same manner, which makes their differentiation challenging. Therefore, this study uses both terms interchangeably. Mood states can be interpreted by constructing profiles that consider normalized values (percentiles) for the following six factors: tension, depression, anger, vigor, fatigue, and confusion. Six mood state profiles were identified: iceberg, inverted iceberg, inverted Everest, fin, surface, and submerged (Parsons-Smith et al., 2017). The same six clusters were found in a study of Brazilian athletes (Brandão et al., 2021).

Emotions and stress are related. Stress is a psychological state comprising emotional and cognitive responses that interfere with the performance of a target behavior (Rosenbaum, 1989). Stress does not reside in the individual or environment but in the relationship between the two; it is not necessarily debilitating and can even facilitate performance (Hanton et al., 2005). Thus, the “directional perception” of stress sources, that is, the individual interpretation of these sources regarding their positive or negative effects, assumes importance (Brandão et al., 2021). Elevated levels of perceived stress can increase competitive anxiety in athletes and potentially worsen their performance (Hammermeister and Burton, 2001). This finding suggests the need to understand the emotions and stress experienced by athletes at different times of competition and their effects on sports performance. In general, athletes perceive that with an increase in the importance of competition, the pressure for performance also increases, generating a great stress load that needs to be managed (Hanton et al., 2005).

Self-control is essential for managing stressful situations. Self-control is an individual’s ability to overcome impulses, temptations, and desires (Hagger, 2013; Englert, 2016) and regulate thoughts and behaviors to achieve long-term goals (Allom et al., 2016). Studies have shown that athletes have higher levels of self-control than the general population (Toering and Jordet, 2015), and individuals with higher levels of self-control perform better in sports tasks where hitting a target is required (Bresin et al., 2012), suggesting that self-control can be trained (Muraven, 2010) and can be an essential psychological characteristic for horizontal jumpers who need to hit the board.

**Figure 1:**
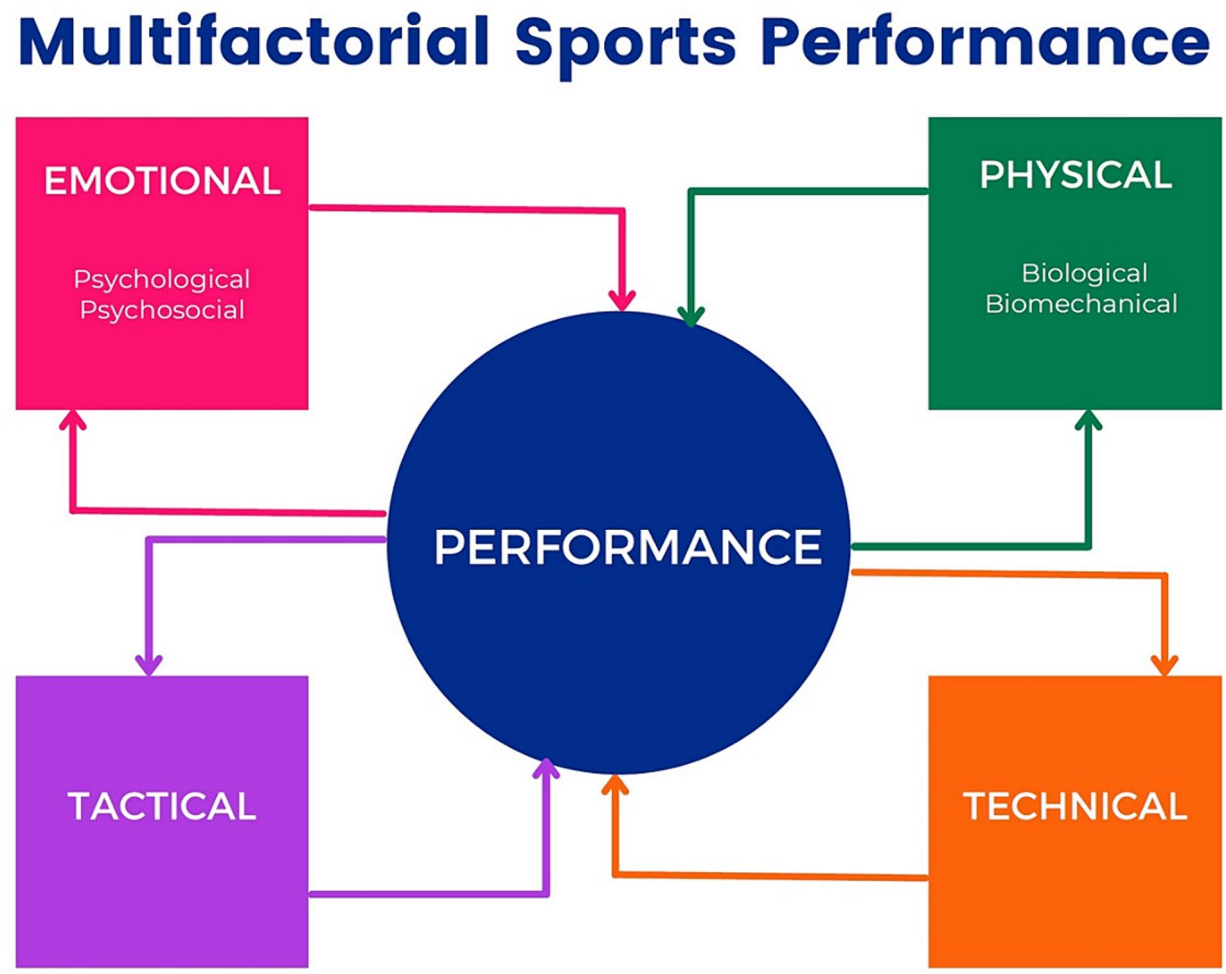
Multifactorial sports performance. Adapted from Brandão and Figueira Junior (1996).

Evidently, sports performance is multifactorial, involving physical components (biological and biomechanical), emotional aspects (psychological and psychosocial), and learning elements (technical and tactical) (Brandão and Figueira Junior, 1996). Therefore, connections between these distinct aspects must be studied (Figure 1).

This study investigated the relationship between psychological factors (emotional regulation, self-control, mood states, and perceived stress) and elements contributing to run-up variability in horizontal jumps. Additionally, this study aimed to conduct comparisons based on sex, events (long jump and triple jump), and contextual situations (training versus competition).

We hypothesized that individuals with elevated self-control and emotional regulation levels would demonstrate enhanced speed, reduced run-up variability, and an earlier onset of the adjustment phase. Conversely, individuals experiencing negative mood states and elevated stress levels were predicted to exhibit decreased speed and increased run-up variability. Furthermore, we anticipated that both events would share comparable psychological and run-up characteristics, whereas sex differences would manifest as distinct psychological traits. Additionally, we hypothesized that there would be significant disparities in psychological constructs and run-up variability between the training and competitive contexts.

## 2 Methods

### 2.1 Study design

This study is a quantitative and observational field research (Gil, 2002), submitted to the Ethics Committee in Research at São Judas University and approved under number CAAE: 40826120.6.0000.0089.

### 2.2 Participants

Ten athletes participated in the study: five males (height: 185.32 ± 6.22 cm; body mass: 81.60 ± 7.23 kg) and five females (height: 166.86 ± 6.72 cm; body mass: 58.20 ± 6.92 kg), with a mean age of 27.14 (±4.25) years, specialists in the long jump and triple jump. The athletes had 13.10 (±3.48) years of experience in athletics and the following mean of personal best results: 6.59 ± 0.21 m, *n* = 2 (female long jumpers); 7.91 ± 0.13 m, *n* = 2 (male long jumpers); 13.09 ± 1.09 m, *n* = 3 (female triple jumpers); and 16.85 ± 0.05 m, *n* = 3 (male triple jumpers). Participants were chosen for convenience and for standing out nationally (among the top ten in the national ranking or in the Brazilian championships in the previous year). Injured athletes who had been away from training and competitions for more than a month were excluded from the sample.

### 2.3 Instruments and procedures

Written informed consent was obtained from all participants, and the study procedures followed the Declaration of Helsinki guidelines. All steps were performed to ensure the athletes’ anonymity and data confidentiality. However, due to the participants’ characteristics, it was not possible to guarantee that they would not be identified. Training sessions and competitions were assessed during a 5-week competition block.

#### 2.3.1 Psychological aspects

**Figure 2:**
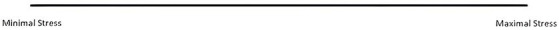
Visual analog scale (VAS) for stress.

To assess psychological aspects (emotional regulation, self-control, mood states, and perceived stress), three questionnaires and a visual analog scale were used, which were answered before the start of training sessions and before warming up for competitions:

– Emotional Regulation Questionnaire (Gross and John, 2003), validated for athletes by Uphill et al. (2012) and for the Brazilian population by Gouveia et al. (2018).
– Brief Self-Control Scale (BSCS) (Tangney et al., 2004), validated for the Brazilian population by Figueira et al. (2019).
– Brunel Mood Scale (BRUMS), validated for Brazilian athletes by Rohlfs & Miranda (De Rohlfs and Miranda, 2006).
– Visual Analog Scale (VAS) for Stress: This instrument is a quick and simple tool for assessing stress levels, consisting of a horizontal line 10 cm long labeled at its ends with “minimal stress” and “maximal stress” (Guimarães, 1998; Gould et al., 2001). Athletes made a mark on the scale indicating how they perceived their level of negative stress at that moment. The distance from the start of the scale to this mark was measured using a ruler, and the value in centimeters represented the perceived level of stress (Figure 2).

The questionnaires were administered in small groups, separated by sex and event, while respecting training and competition schedules. The questionnaires were distributed 30 min before the start of training sessions and 90 min before the competitions to avoid interference with the warm-up and athletes’ presentations before entering the track.

#### 2.3.2 Run-up variability

Run-up variability was evaluated in two situations: during technical training sessions, where athletes performed run-ups followed by take-off without landing, and during official competitions. A total of 98 run-ups from four training sessions and 129 jumps from four competitions were analyzed. Each athlete participated in at least two data collection procedures for each condition.

##### 2.3.2.1 Training sessions

**Figure 3:**
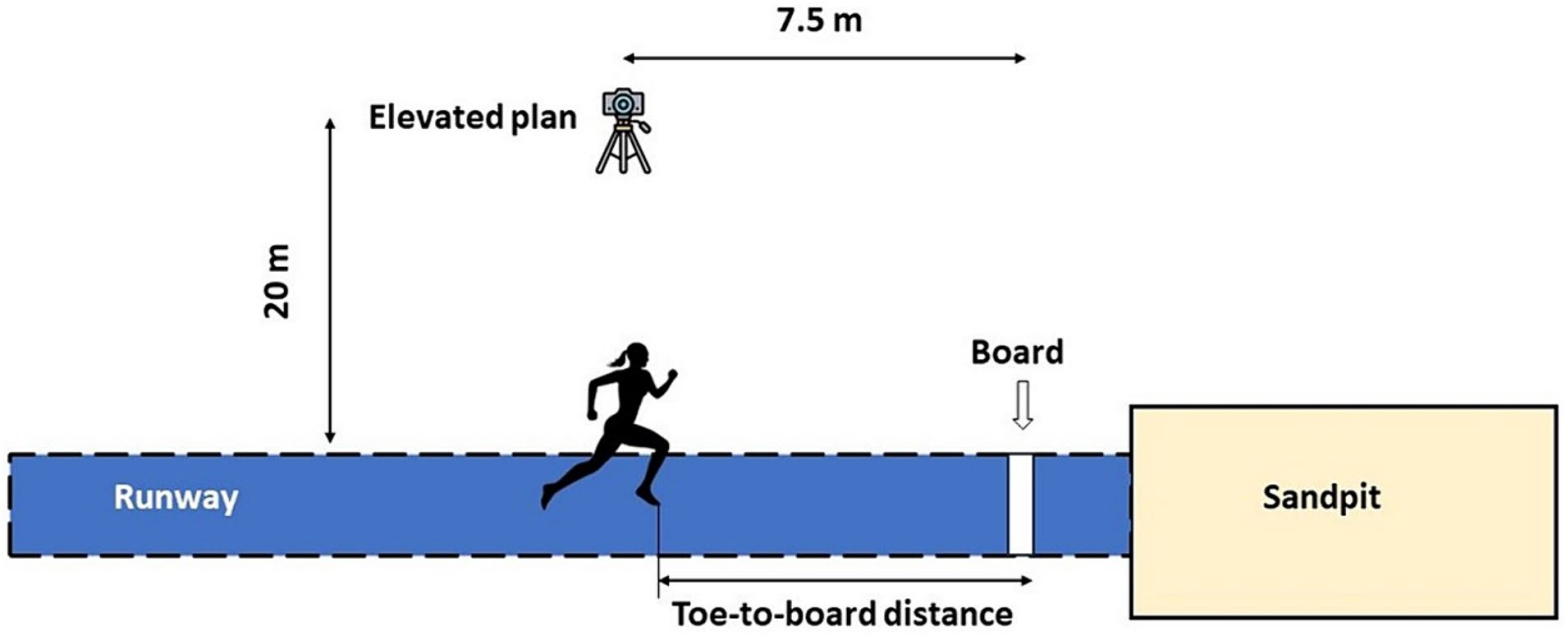
Procedures for measuring run-up variability. Adapted from Theodorou et al. (2013).

The athletes performed four attempts with approximately 5 min of rest between them. The pre-training warm-up routine was the same as that the athletes used (jogging, dynamic stretching, running or jumping drills, and accelerations) and lasted approximately 30 min. Marks were made with adhesive tape on both sides of the runway at every meter for later analysis using Dartfish 10 Team ProData software (Dartfish, Fribourg, Switzerland). Jumps were recorded on HD video at 120 frames per second with a Sony FDR-AX53 camera (Sony Electronics Inc., San Jose, CA, USA), which was fixed on a tripod 20 meters from the jump runway, 7.5 meters before the takeoff board, eight meters above the level of the track (Figure 3). The average speed in the final segment of the run-up was evaluated by positioning two sets of Witty dual-beam photocells (Microgate, Bolzano, Italy) alongside the runway; one pair was positioned at 1 meter and the other at 6 meters before the take-off board.

The protocol for assessing run-up variability is commonly used in athletics. In a series of jumps or run-ups, the toe-to-board distance is measured along the entire run-up (Lee et al., 1982; McCosker et al., 2020) or a certain number of steps before take-off (Theodorou et al., 2013; Makaruk et al., 2015). For each event (training or competition), the standard deviation of this distance for each step is calculated, indicating the accumulated error in that step. Our study measured running variability in the last six steps (Moura et al., 2023).

##### 2.3.2.2 Competition sessions

The same procedures as in the training sessions were followed, with the athletes performing three to six attempts at each competition.

For each training and competition event, the following parameters were used to evaluate the run-up variability:

- Distance lost at the board: the distance between the take-off point and the measuring line, with negative values indicating failed attempts.
- Percentage of failed attempts.
- The magnitude of the largest accumulated error: the highest standard deviation was found in the last six steps of the run-up.
- The adjustment onset: the step where the largest error was observed, representing the onset of the visual regulation and adjustment phases.
- Absolute adjustment: the difference between the largest accumulated error and the error observed on the board.
- Relative adjustment: the percentage difference between the largest accumulated error and the error observed on the board.
- Average approach speed.

### 2.4 Statistical analysis

Data analysis was performed using JASP 0.16.1 software (University of Amsterdam, Netherlands). The Shapiro–Wilk test indicated a normal distribution, and the mean and standard deviation were calculated. The independent samples t-test was used to compare sexes and events. Paired samples t-test was used to compare the competition and training situations. Additionally, effect size (ES) was calculated for all differences using Cohen’s *d*, with the following interpretation criteria: *d* ≤ 0.19 = insignificant; 0.20 ≤ *d* ≤ 0.49 = small; 0.50 ≤ *d* ≤ 0.79 = medium; 0.80 ≤ *d* ≤ 1.29 = large; *d* ≥ 1.30 = very large (Espírito-Santo and Daniel, 2015). Relationships between variables were quantified using the Pearson’s correlation coefficient. The significance level was set at *p* < 0.05.

## 3 Results

Athletes’ *t*-tests for independent and paired samples were used to identify differences between events, situations, and sexes.

### 3.1 Comparisons between events

Table 1 presents the outcomes of independent samples t-tests comparing psychological constructs between the events in both training and competition, without considering the participants’ sexes. Emotional regulation showed a significant difference (*p* = 0.01), with higher scores observed for the long jump.

**Table 1:** Comparison of psychological constructs between events, across all situations, and both sexes.

	**Long Jump Mean ± SD**	**Triple Jump Mean ± SD**	***p***	**ES**
Self-control (score)	34.74 ± 5.28	33.78 ± 4.53	0.67	0.20
Stress (cm)	4.07 ± 1.98	3.04 ± 2.16	0.30	0.49
Emotional Regulation (score)	53.02 ± 5.68	46.00 ± 5.73	0.01**	1.23++
Tension (score)	3.43 ± 2.06	2.71 ± 2.63	0.52	0.30
Depression (score)	0.35 ± 0.35	0.47 ± 0.53	0.61	−0.24
Anger (score)	1.30 ± 1.87	1.22 ± 1.61	0.92	0.05
Vigor (score)	10.88 ± 2.70	10.57 ± 2.59	0.80	0.12
Fatigue (score)	2.57 ± 1.91	1.79 ± 1.86	0.38	0.42
Confusion (score)	1.36 ± 1.23	1.33 ± 1.91	0.97	0.02

**p < 0.01, ++ ES large.ES, Effect size (Cohen’s *d*).

Table 2 displays the results of independent samples t-tests comparing aspects of run-up variability between the events in training and competition without distinguishing by sex. No significant differences were found, although the long jump demonstrated higher values for speed with a medium effect size.

**Table 2:** Comparison of aspects of run-up variability between events, across all situations, and both sexes.

	**Long Jump Mean ± SD**	**Triple Jump Mean ± SD**	***p***	**ES**
Board losses (m)	0.03 ± 0.05	0.00 ± 0.10	0.42	0.38
Failed attempts (%)	50.16 ± 14.41	58.49 ± 22.89	0.37	−0.42
Speed (m/s)	9.55 ± 0.51	9.01 ± 0.80	0.11	0.77+
Largest error (m)	0.35 ± 0.09	0.40 ± 0.18	0.51	−0.31
Adjustment onset (n)	4.62 ± 0.91	4.21 ± 0.97	0.36	0.43
Absolute adjustment (m)	0.21 ± 0.05	0.21 ± 0.10	0.83	−0.10
Relative adjustment (%)	59.17 ± 8.02	55.87 ± 13.52	0.54	0.28

+ ES medium.ES, effect size (Cohen’s *d*).

### 3.2 Comparisons between sexes

Table 3 shows the outcomes of the independent samples t-tests comparing psychological constructs between the sexes in both training and competition. Significant differences were found for tension (p = 0.01) and depression (*p* < 0.001), with females exhibiting higher values. Medium effect sizes were observed for stress, anger, and confusion, all of which were higher among females. Conversely, males displayed higher vigor scores with a medium effect size.

**Table 3:** Comparison of psychological constructs between sexes in horizontal jumps, across all situations.

	**Female Mean ± SD**	**Male Mean ± SD**	***p***	**ES**
Self-control (score)	33.90 ± 2.88	34.42 ± 6.23	0.81	−0.11
Stress (cm)	4.17 ± 1.79	2.73 ± 2.23	0.13	0.72+
Emotional regulation (score)	50.00 ± 6.96	47.62 ± 6.33	0.43	0.36
Tension (score)	4.30 ± 2.43	1.69 ± 1.52	0.01**	1.29++
Depression (score)	0.75 ± 0.42	0.09 ± 0.18	<0.001***	2.02+++
Anger (score)	1.83 ± 2.01	0.68 ± 1.04	0.12	0.72+
Vigor (score)	9.80 ± 1.64	11.59 ± 3.08	0.12	−0.73+
Fatigue (score)	2.12 ± 1.78	2.09 ± 2.06	0.98	0.01
Confusion (score)	1.77 ± 1.96	0.93 ± 1.18	0.26	0.52+

**p < 0.01, ***p < 0.001, + ES medium, ++ ES large, +++ ES very large.ES, effect size (Cohen’s *d*).

Table 4 shows the results of independent sample t-tests comparing aspects of run-up variability between the sexes in both training and competition. A significant difference was found for speed (p < 0.001), with males displaying greater values. Additionally, females showed higher losses on the board with a medium effect size.

**Table 4:** Comparison of aspects of run-up variability between sexes in horizontal jumps, across all situations.

	**Female Mean ± SD**	**Male Mean ± SD**	***p***	**ES**
Board losses (m)	0.04 ± 0.06	−0.01 ± 0.10	0.16	0.65+
Failed attempts (%)	52.86 ± 17.06	57.46 ± 23.16	0.62	−0.23
Speed (m/s)	8.60 ± 0.48	9.86 ± 0.17	< 0.001***	−3.51+++
Largest error (m)	0.36 ± 0.08	0.40 ± 0.20	0.64	−0.21
Adjustment onset (n)	4.53 ± 1.02	4.21 ± 0.88	0.46	0.34
Absolute adjustment (m)	0.20 ± 0.06	0.22 ± 0.10	0.70	−0.17
Relative adjustment (%)	56.39 ± 8.89	57.99 ± 14.10	0.76	−0.14

***p < 0.001, + ES medium, +++ ES very large.ES, effect size (Cohen’s *d*).

### 3.3 Comparisons between training and competition

Table 5 displays the results of the paired samples *t*-tests comparing psychological constructs between training and competition. Significant differences were observed for vigor (*p* = 0.01), which was higher in competition, fatigue (*p* < 0.001), and confusion (*p* = 0.02), both of which were higher in training. Additionally, there was a medium effect size for anger, which was higher in training.

**Table 5:** Comparison of psychological constructs between training and competition situations in horizontal jumps across both sexes.

	**Training Mean ± SD**	**Competition Mean ± SD**	***p***	**ES**
Self-control (score)	34.70 ± 4.84	33.63 ± 4.82	0.30	0.35
Stress (cm)	3.37 ± 1.97	3.53 ± 2.33	0.82	−0.08
Emotional Regulation (score)	48.77 ± 6.96	48.85 ± 6.57	0.94	−0.02
Tension (score)	2.62 ± 2.46	3.38 ± 2.38	0.27	−0.37
Depression (score)	0.48 ± 0.41	0.36 ± 0.52	0.43	0.26
Anger (score)	1.93 ± 2.02	0.57 ± 0.88	0.06	0.68+
Vigor (score)	9.72 ± 2.68	11.68 ± 2.15	0.01**	−1.02++
Fatigue (score)	3.18 ± 1.67	1.02 ± 1.42	< 0.001***	1.28++
Confusion (score)	1.82 ± 1.83	0.88 ± 1.34	0.02*	0.91++

*p < 0.05, **p < 0.01, ***p < 0.001, + ES medium, ++ ES large.ES, effect size (Cohen’s *d*).

Table 6 presents the results of the paired *t*-test comparing aspects of run-up variability between training and competition. A significant difference was found for failed attempts (*p* = 0.02), which were higher in training. Furthermore, medium effect sizes were observed for board losses and speed, both of which were higher in competition.

**Table 6:** Comparison of aspects of run-up variability between training and competition situations in horizontal jumps across both sexes.

	**Training Mean ± SD**	**Competition Mean ± SD**	***p***	**ES**
Board losses (m)	−0.02 ± 0.10	0.04 ± 0.04	0.07	−0.65+
Failed attempts (%)	66.53 ± 16.86	43.79 ± 16.35	0.02*	0.92++
Speed (m/s)	9.20 ± 0.77	9.26 ± 0.73	0.15	−0.50+
Largest error (m)	0.43 ± 0.18	0.33 ± 0.09	0.20	0.44
Adjustment onset (n)	4.47 ± 1.00	4.28 ± 0.93	0.52	0.21
Absolute adjustment (m)	0.23 ± 0.10	0.19 ± 0.05	0.32	0.33
Relative adjustment (%)	57.23 ± 14.90	57.15 ± 7.55	0.99	0.01

*p < 0.05, + ES medium, ++ ES large.ES, effect size (Cohen’s *d*).

### 3.4 Relationship between variables

The *t*-tests revealed non-significant differences across most psychological constructs and aspects of run-up variability when comparing the events, except emotional regulation. Therefore, all athletes were treated as participants in horizontal jumps for the correlation analysis, without distinguishing between specific events.

Significant differences were observed across more variables when comparing situations (training and competition) and sex (male and female). Therefore, separate analyses were conducted for different situations and sexes.

Correlation matrices were generated for each condition, highlighting significant relationships in bold. Table 7 shows the correlation matrix for female athletes during training, revealing statistically significant correlations between the eight pairs of variables.

**Table 7:** Correlation matrix between psychological constructs and aspects of run-up variability in horizontal jumps for females, in training.

	**Self-control**	**Stress**	**Emotional regulation**	**Tension**	**Depression**	**Anger**	**Vigor**	**Fatigue**	**Confusion**	**Board losses**	**Failed attempts**	**Speed**	**Largest error**	**Adjust. onset**	**Absolute adjustment**	**Relative adjustment**
1.Self-control	—															
2.Stress	0.63	—														
3.Emotional regulation	−0.04	0.74	—													
4.Tension	0.73	0.43	−0.02	—												
5.Depression	−0.62	−0.58	−0.35	−0.73	—											
6.Anger	0.77	0.34	−0.21	0.95*	−0.53	—										
7.Vigor	0.36	0.26	−0.01	−0.36	0.05	−0.28	—									
8.Fatigue	0.85	0.61	0.11	0.97**	−0.81	0.91*	−0.16	—								
9.Confusion	0.94*	0.38	−0.28	0.61	−0.57	0.66	0.48	0.71	—							
10.Board losses	0.52	0.66	0.42	0.85	−0.62	0.76	−0.48	0.84	0.24	—						
11.Failed attempts	0.16	0.02	−0.18	−0.54	0.34	−0.40	0.95*	−0.38	0.31	−0.67	—					
12.Speed	−0.44	0.06	0.39	0.05	0.25	0.02	−0.76	−0.07	−0.71	0.46	−0.69	—				
13.Largest error	0.64	0.32	−0.01	0.43	−0.80	0.30	0.42	0.55	0.78	0.10	0.19	−0.77	—			
14.Adjustment onset	0.42	0.55	0.22	−0.19	0.22	−0.03	0.74	−0.01	0.31	−0.05	0.72	−0.20	−0.05	—		
15.Abs. adjustment	0.40	0.37	0.25	0.04	−0.64	−0.12	0.63	0.22	0.53	−0.13	0.41	−0.76	0.89*	0.12	—	
16.Relative adjustment	0.04	0.37	0.51	−0.37	−0.32	−0.53	0.68	−0.17	0.12	−0.32	0.53	−0.52	0.54	0.28	0.86	—

*p < 0.05, **p < 0.01, ***p < 0.001.

Table 8 illustrates the correlation matrix for female athletes during the competition, demonstrating statistically significant correlations between the 11 pairs of variables.

**Table 8:** Correlation matrix between psychological constructs and aspects of run-up variability in horizontal jumps for females, in competition.

	**Self-control**	**Stress**	**Emotional Regulation**	**Tension**	**Depression**	**Anger**	**Vigor**	**Fatigue**	**Confusion**	**Board losses**	**Failed attempts**	**Speed**	**Largest error**	**Adjust. onset**	**Absolute adjustment**	**Relative adjustment**
1.Self-control	—															
2.Stress	0.31	—														
3.Emotional Regulation	0.45	−0.32	—													
4.Tension	0.18	0.68	−0.72	—												
5.Depression	−0.35	0.65	−0.90*	0.82	—											
6.Anger	0.11	0.86	−0.52	0.87	0.81	—										
7.Vigor	0.36	0.40	−0.45	0.45	0.32	0.15	—									
8.Fatigue	0.40	0.84	−0.23	0.79	0.56	0.94*	0.09	—								
9.Confusion	0.43	0.84	−0.31	0.85	0.60	0.94*	0.20	0.99**	—							
10.Board losses	0.00	0.32	−0.86	0.76	0.69	0.40	0.78	0.23	0.35	—						
11.Failed attempts	−0.87*	0.01	−0.43	0.07	0.52	0.29	−0.54	0.05	−0.01	−0.09	—					
12.Speed	−0.21	−0.96**	0.55	−0.84	−0.80	−0.92*	−0.48	−0.84	−0.87	−0.54	−0.08	—				
13.Largest error	−0.39	−0.51	−0.48	0.25	0.20	−0.06	−0.14	−0.18	−0.12	0.41	0.30	0.27	—			
14.Adjustment onset	−0.07	0.68	−0.08	0.39	0.49	0.79	−0.39	0.78	0.69	−0.22	0.53	−0.61	−0.30	—		
15.Abs. adjustment	0.01	−0.76	−0.04	−0.11	−0.35	−0.53	0.04	−0.49	−0.42	0.24	−0.27	0.60	0.78	−0.76	—	
16.Relative adjustment	0.45	0.12	0.69	−0.55	−0.57	−0.37	0.15	−0.20	−0.24	−0.49	−0.55	0.12	−0.89*	−0.12	−0.43	—

*p < 0.05, **p < 0.01, ***p < 0.001.

Table 9 presents the correlation matrix for male athletes during training, identifying statistically significant correlations between the 11 pairs of variables.

**Table 9:** Correlation matrix between psychological constructs and aspects of run-up variability in horizontal jumps for males, in training.

	**Self-control**	**Stress**	**Emotional Regulation**	**Tension**	**Depression**	**Anger**	**Vigor**	**Fatigue**	**Confusion**	**Board losses**	**Failed attempts**	**Speed**	**Largest error**	**Adjust. onset**	**Absolute adjustment**	**Relative adjustment**
1.Self-control	—															
2.Stress	−0.56	—														
3.Emotional Regulation	−0.45	0.37	—													
4.Tension	−0.34	0.79	0.76	—												
5.Depression	−0.01	0.69	0.62	0.93*	—											
6.Anger	−0.75	0.48	−0.06	−0.04	−0.21	—										
7.Vigor	−0.30	0.39	0.45	0.33	0.39	0.49	—									
8.Fatigue	−0.36	0.11	0.96**	0.57	0.43	−0.18	0.35	—								
9.Confusion	−0.39	0.57	0.92*	0.93*	0.80	−0.16	0.26	0.82	—							
10.Board losses	0.46	−0.55	0.44	0.07	0.16	−0.83	−0.15	0.61	0.34	—						
11.Failed attempts	−0.78	0.44	−0.20	−0.08	−0.34	0.87	0.02	−0.30	−0.18	−0.85	—					
12.Speed	−0.30	0.83	0.69	0.94*	0.94*	0.13	0.60	0.47	0.80	−0.06	−0.07	—				
13.Largest error	−0.42	−0.49	0.22	−0.41	−0.62	0.22	0.04	0.42	−0.10	0.24	0.21	−0.47	—			
14.Adjustment onset	−0.18	0.72	0.18	0.49	0.60	0.49	0.80	−0.05	0.22	−0.48	0.14	0.75	−0.51	—		
15.Abs.adjustment	−0.62	0.36	−0.30	−0.23	−0.36	0.97**	0.35	−0.40	−0.38	−0.90*	0.88*	−0.05	0.18	0.41	—	
16.Relative adjustment	−0.29	0.80	−0.22	0.32	0.33	0.61	0.29	−0.48	−0.02	−0.86	0.56	0.48	−0.63	0.76	0.62	—

*p < 0.05, **p < 0.01, ***p < 0.001.

Finally, Table 10 presents the correlation matrix for male athletes during competitions, revealing statistically significant correlations between the 13 pairs of variables.

**Table 10:** Correlation matrix between psychological constructs and aspects of run-up variability in horizontal jumps for males, in competition.

	**Self-control**	**Stress**	**Emotional regulation**	**Tension**	**Depression**	**Anger**	**Vigor**	**Fatigue**	**Confusion**	**Board losses**	**Failed attempts**	**Speed**	**Largest error**	**Adjust. onset**	**Absolute adjustment**	**Relative adjustment**
1.Self-control	—															
2.Stress	−0.09	—														
3.Emotional Regulation	−0.46	0.41	—													
4.Tension	0.37	0.83	−0.10	—												
5.Depression	−0.20	−0.58	0.50	−0.84	—											
6.Anger	−0.31	0.88*	0.65	0.59	−0.29	—										
7.Vigor	0.04	0.05	0.07	−0.09	0.10	−0.27	—									
8.Fatigue	−0.16	0.95*	0.62	0.70	−0.35	0.97**	−0.10	—								
9.Confusion	0.01	0.96**	0.55	0.77	−0.39	0.88*	0.10	0.97**	—							
10.Board losses	0.95*	0.04	−0.54	0.49	−0.40	−0.30	0.26	−0.12	0.10	—						
11.Failed attempts	−0.25	−0.24	0.40	−0.57	0.63	−0.30	0.81	−0.23	−0.13	−0.18	—					
12.Speed	−0.27	0.84	0.82	0.43	−0.04	0.85	0.23	0.91*	0.91*	−0.21	0.20	—				
13.Largest error	0.54	0.46	−0.02	0.55	−0.33	0.06	0.72	0.29	0.53	0.72	0.28	0.39	—			
14.Adjustment onset	−0.52	0.70	0.78	0.21	0.03	0.69	0.44	0.73	0.73	−0.39	0.45	0.92*	0.33	—		
15.Abs.adjustment	0.57	0.49	0.10	0.55	−0.24	0.14	0.66	0.37	0.59	0.70	0.28	0.47	0.99**	0.37	—	
16.Relative adjustment	0.49	0.52	0.50	0.50	0.04	0.54	−0.05	0.64	0.71	0.38	−0.10	0.62	0.48	0.30	0.62	—

*p < 0.05, **p < 0.01, ***p < 0.001.

## 4 Discussion

This study investigated the relationship between run-up variability in horizontal jumps and various psychological constructs, with comparisons based on sex, event type, and context (training versus competition). The findings indicate that the events were similar in terms of both technical and psychological aspects. However, significant differences emerged when comparing sexes and situational contexts. Negative mood states were interrelated, and female athletes who exhibited higher levels of emotional regulation experienced lower levels of depression. Additionally, higher levels of self-control were associated with fewer failed attempts.

### 4.1 Comparisons between events

When we compared the events, only emotional regulation showed a significant difference in favor of the long jump. This similarity was expected since these events are classified within the same subgroup called “horizontal jumps” in athletics (Hay, 1993), with performance being determined by similar factors such as approach speed (Moura et al., 2005b), special strength (Moura and de Paula Moura, 2001), and precision on the board (Hay and Koh, 1988). It is common to find athletes who participate with similar levels of success in both events. Although data were collected only for the main event in the studied group, various athletes competed in both the long and triple jumps. This justifies the analysis of the relationships between the variables, considering that all athletes integrated into the same group (horizontal jumps) without distinction regarding events. Despite this, the difference in speed, although not statistically significant, presented medium effect sizes, and this finding will be discussed below.

The run-up speed was higher in the long jump. It has long been discussed whether faster athletes are preferentially selected for the long jump or whether the characteristics of the triple jump event limit the possibility of using a larger portion of the maximum speed capacity (Hay, 1993). The relationship between approach speed and performance in both jumps is positive and significant. However, the correlation values are higher in the long jump (Moura et al., 2005a), suggesting a more significant number of determining factors in the triple jump (Hutt, 1989; Moura et al., 2023). Hay (1993) demonstrated that when studying athletes who participated in both events, approach speeds were higher in the long jump, which strengthens the understanding that it is the characteristics and demands of the events and not necessarily the athletes’ abilities that are responsible for the reduced values of approach speed in the triple jump.

### 4.2 Comparisons between sexes

Significant differences were found between the sexes. Among psychological constructs, depression, and tension were higher among females. Additionally, medium effect sizes were found for confusion, stress and anger, which were higher in females, and vigor, which was higher in males. Differences in mood states between male and female athletes have been noted previously, and the same trends were verified in this study. Our results are similar to those found by Brandt et al. (2010) when studying Brazilian sailors, in which women had higher values of stress, depression, and anger, and lower vigor than men. In a study of 953 young Brazilian athletes, Brandão et al. (2021) found higher values for negative mood states, such as fatigue, confusion, and depression, among women, who also had a higher prevalence of the inverted iceberg and inverted Everest clusters. Han et al. (2021), when validating the Brunel Mood Scale in the cultural context of Singapore, found that mood scores varied predictably when participants were divided into distinct groups, particularly when separated by sex, where women had higher values for depression, anger, stress, fatigue, and confusion, and men hd higher vigor. A similar study among the Spanish population had comparable results, with women having higher scores for anger, depression, confusion, and fatigue and men having higher scores for vigor (Cañadas et al., 2017). Brandt et al. (2011) point to the psychophysiological characteristics of females to explain the higher levels of anger typically found among women. However, the possibility that these differences were partially caused by sociocultural factors cannot be ignored. Women were belatedly admitted into sports in the fight for equity that crossed the 20th century and is ongoing (Rubio and Veloso, 2019). This may increase demand beyond what is implicit within the sport, leading to fear of failure, perfectionism, and concerns about body image, among other factors (Killham et al., 2018), potentially altering mood states and emotions (Fox, 2008).

Regarding the components of run-up variability, speed was significantly higher among males, as expected. The average approach speed of female jumpers (8.60 ± 0.48 m/s) represented 87.22% of that of male jumpers (9.86 ± 0.17 m/s), a percentage similar to that observed in major international competitions. For example, analyzing the reports from the 2009 World Championships in Berlin, where average speeds measured in the same way as in our study were reported, men approached the board at 10.42 ± 0.21 m/s in their best trials in the long jump final and 10.05 ± 0.22 m/s in the triple jump final. However, women had speeds of 9.28 ± 0.32 m/s in the long jump final and 8.97 ± 0.22 m/s in the triple jump final, respectively 89.09 and 89.25% of the values presented by men (Mendoza et al., 2009a,b).

### 4.3 Comparisons between training and competition

Significant differences were observed between the training and competition situations. Among the psychological constructs, fatigue and confusion presented higher values in training, whereas vigor was higher in competition. Fatigue was expected to be lower on competition days, as this is a condition for achieving superior results. In the days leading up to the most important competitions, it is common to adopt tapering practices, in which the training load is substantially reduced to eliminate the effects of fatigue and allow for the expression of adaptations caused by training (Mujika, 2009). This procedure may also be related to increased vigor during competitions, which has been negatively associated with fatigue (Rohlfs et al., 2008). Prapavessis & Grove (Prapavessis and Grove, 1994) evaluated shooters at four-time points before the competition (from 48 h to 15 min before its start) and noticed an acute increase in vigor 15 min before the event. This study also found larger effect sizes for anger, during training. These differences seen together, moved in the desired direction, favoring the manifestation of the iceberg profile during competitions, a profile considered to facilitate performance (Lochbaum et al., 2021).

Among the aspects of run-up variability, there were significantly more failed attempts in training, as also noted by McCosker et al. (2020). Although this may be related to the absence of consequences for fouls in this situation, these authors believe that experienced athletes are able to use the information provided by the competition environment (for example, the position of the referee next to the take-off board) as an additional reference to adjust step lengths, information that is not available during training. Medium effect sizes were observed for losses on the board and speed, both higher in competitions, which may reinforce their importance for performance in competitions. Approach speed is considered a critical factor in determining jumping distance (Moura et al., 2005b). During competitions, athletes may be more motivated and in a heightened state of alertness, which can lead to an increase in run-up speed compared to training and, consequently, improved performance.

### 4.4 Relationship between variables

In general, among both sexes, during training and competition, negative mood states such as depression, tension, anger, fatigue, and confusion were related to each other and to stress. This finding confirms those of other authors studying the relationship between stress factors, dimensions of burnout syndrome, and negative mood states, with positive associations in all these aspects (Rinaldi, 2020).

It was expected that individuals with higher emotional regulation and self-control would better manage their mood states, as in Tamir et al.’s study (Tamir et al., 2008), where individuals used emotional regulation strategies to evoke the mood state that best fits the task at hand, but overall, this hypothesis was not confirmed in the present study. Nevertheless, females with higher emotional regulation in competition showed lower levels of depression, an important finding because this mood state tends to elevate the values of other negative states, modulating their deleterious influence on performance (Lane et al., 2005). In turn, self-control showed a strong relationship with losses on the board in competition for males (0.95, *p* < 0.05), which, considering the presented data, can be seen as positive, as it could decrease the number of failed attempts. In competition, the relationship between self-control and failed attempts among females was high (−0.87, *p* = 0.05), acting precisely in that direction. Findings from Bresin et al. (2012), who demonstrated that individuals with more self-control hit the target more accurately in computerized tasks, and Rosenbaum (1989), who suggested that coping with stress-generating situations, such as the need to produce valid jumps in competitions, requires self-control, support our results.

## 5 Limitations

Although it is a common characteristic in investigations of high-performance athletes, the small number of participants represents a limitation of our study, as it makes it difficult to generalize the conclusions and reduces the statistical power to identify differences where they really exist. Furthermore, in our experimental model, we used the traditional approach to study run-ups in horizontal jumps, where there is a growing variability in the first part of the run-up, followed by a decrease from six to four steps before the take-off board (Hay, 1993). Therefore, we measured variability only in the last six steps because this procedure allowed us to extract the most critical indicators. Recently, it was demonstrated that Australian international-level athletes have greater functional variability in the first part of the run-up and start visual regulation very early, up to 17 steps before the board, unlike national-level athletes, who would behave similarly to the traditional model (McCosker et al., 2020). Therefore, the fact that we did not evaluate the first part of the run-up constitutes a limitation of our study. Due to logistical constraints and to avoid interference with each athlete’s preparation ritual, the evaluation of psychological constructs occurred a considerable amount of time before the start of the competitions (90 min), and there was no attempt to measure changes during the competition or training. The measurement of heart rate variability, whose parasympathetic variation is related to vigor (Leite et al., 2013), may help estimate mood changes during competition when researchers usually do not have contact with athletes, and it is possible to explore this in the future.

## 6 Conclusion

Long jump and triple jump exhibit comparable psychological and run-up variability characteristics. This similarity indicates that strategies for emotional regulation, speed, and run-up accuracy optimization may broadly apply across both events. However, notable differences were found between female and male jumpers in terms of their emotional states, implying that distinct strategies should be employed to promote optimal mood states during competitions, with a particular focus on utilizing emotional regulation tools to modulate depression among female jumpers.

The higher number of failed attempts in training suggests that the strategies used may not replicate the demands of the competition or effectively facilitate the desired changes as intended by coaches. Therefore, it is crucial to explore training run-up methods to simulate the competition’s context and demands more closely.

The onset of competition adjustment is associated with increased losses on the board, which, in turn, inversely correlates with the number of failed attempts. If these losses remain within appropriate values, exploring the ability to initiate the adjustment phase early is essential.

Among the psychological constructs, self-control emerged as a significant factor associated with fewer failed attempts or greater losses on the board, both of which can contribute to a more successful performance. Hence, integrating techniques for developing self-control should be considered in the preparation of horizontal jumpers.

Although this study provides insights into the interplay between psychological constructs and run-up variability in horizontal jumps, there are still areas for further research to explore. Future investigations could consider the entire course of the run-up and the emotional changes that occur leading up to and during the competition. Such studies could enhance our understanding of the complex inter-relationships between psychological constructs and run-up variability during horizontal jumps. This study represents a preliminary step in this direction and contributes to the growing body of knowledge in this area.

## 7 Practical recommendations

- Incorporate Emotional Regulation Training: Develop training sessions that focus on emotional regulation techniques such as self-talk, relaxation, and visualization. These can help athletes manage stress and maintain optimal mood states.
- Simulate Competition Conditions in Training: Create training scenarios that mimic the pressures and conditions of competition. This can help athletes adapt their run-up strategies and reduce the number of failed attempts during actual competitions.
- Focus on Early Run-Up Adjustments: Encourage athletes to start their run-up adjustments earlier to maintain speed and improve accuracy at the take-off board. This approach can help reduce variability and increase performance consistency.
- Enhance Self-Control Skills: Implement exercises and routines aimed at improving self-control, which has been linked to fewer failed jumps. Techniques may include goal setting, impulse control exercises, and mental rehearsal.
- Tailored Training Based on Sex Differences: Recognize and address the distinct psychological needs of male and female athletes. For instance, additional support for emotional regulation may be necessary for female athletes to manage higher levels of depression and tension.

## Data availability statement

The raw data supporting the conclusions of this article will be made available by the authors, without undue reservation.

## Ethics statement

The studies involving humans were approved by Ethics Committee in Research at São Judas University and approved under number CAAE: 40826120.6.0000.0089. The studies were conducted in accordance with the local legislation and institutional requirements. The participants provided their written informed consent to participate in this study.

## Author contributions

LPM: Conceptualization, Formal analysis, Methodology, Writing – original draft, Writing – review & editing. NAM: Writing – original draft, Writing – review & editing, Conceptualization, Methodology. TFPM: Writing – review & editing. TBMAM: Writing – review & editing. MRFB: Conceptualization, Formal analysis, Methodology, Writing – review & editing.
